# Di­aqua­bis­{μ-1,5-bis­[(pyridin-2-yl)methyl­idene]carbonohydrazide(1–)}di-μ-chlorido-tetra­chlorido­tetra­zinc(II)

**DOI:** 10.1107/S2056989020009834

**Published:** 2020-07-24

**Authors:** Thierno Moussa Seck, Papa Aly Gaye, Cheikh Ndoye, Ibrahima Elhadji Thiam, Ousmane Diouf, Pascal Retailleau, Mohamed Gaye

**Affiliations:** a Département de Chimie, Faculté des Sciences et Techniques, Université Cheikh Anta Diop, Dakar, Senegal; b Département de Chimie, UFR Sciences et Techniques, Université Assane Seck, Ziguinchor, Senegal; c Substances Naturelles, CNRS UPR 2301, Université Paris-Sud, Université, Paris-Saclay, 1 av. de la Terrasse, 91198 Gif-sur-Yvette, France

**Keywords:** zinc, complex, crystal structure, tetra­nuclear, square-pyramidal coordination

## Abstract

A tetra­nuclear Zn^II^ complex, [Zn_4_(C_13_H_11_N_6_O)_2_Cl_6_(H_2_O)_2_] or {[Zn_2_(HL)(H_2_O)(Cl_2_)](μCl)_2_[Zn_2_(HL)(H_2_O)(Cl)]}_2_, was synthesized by mixing an equimolar amount of a methanol solution containing ZnCl_2_ and a methanol solution containing the ligand H_2_ *L* [1,5-bis­(pyridin-2-yl­methyl­ene)carbono­hydrazide]. In the tetra­nuclear complex, each of the two ligand mol­ecules forms a dinuclear unit that is connected to another dinuclear unit by two bridging chloride anions. In each dinuclear unit, one Zn^II^ cation is penta­coordinated in a N_2_OCl_2_ in a distorted square-pyramidal geometry, while the other Zn^II^ cation is hexa­coordinated in a N_3_OCl_2_ environment with a distorted octa­hedral geometry. The basal plane around the penta­coordinated Zn^II^ cation is formed by one chloride anion, one oxygen atom, one imino nitro­gen atom and one pyridine nitro­gen atom with the apical position occupied by a chloride anion. The basal plane of the hexa­coordinated Zn^II^ cation is formed by one chloride anion, one hydrazinyl nitro­gen atom, one imino nitro­gen atom and one pyridine nitro­gen atom with the apical positions occupied by a water oxygen atom and a bridged chloro anion from another dinuclear unit, leading to a tetra­nuclear complex. A series of intra­molecular C—H⋯Cl hydrogen bonds is observed in each tetra­nuclear unit. In the crystal, the tetra­nuclear units are connected by inter­molecular C—H⋯Cl, C—H⋯O and N—H⋯O hydrogen bonds, forming a planar two-dimensional structure in the *ac* plane.

## Abstract 2

A symmetrical dicarbonohydrazide was used to synthesize a centrosymmetric tetra­nuclear zinc(II) complex in which two of the zinc cations are penta­coordinated and the other two are hexa­coordinated.

## Chemical context   

Symmetrical dicarbonohydrazide Schiff bases possess two cavitiess, which make them versatile. During complexation, either one or both of the cages can be occupied by a metal ion depending on the reaction conditions. The presence of an amidic bond in these mol­ecules leads to the keto-enol tautomer, which can act in neutral or deprotonated forms. These compounds can adopt two different configurations, *e.g. S-cis* or *S-trans*, yielding different structures with the same metal cation. These ligands can coordinate to transition metals in a penta­dentate or hexa­dentate manner (El-Gammal *et al.*, 2012 ▸; Sow *et al.*, 2013 ▸), as well as in the ketonic or enolic form (Zhang *et al.*, 2014 ▸). When the configuration of this kind of ligand is *S-trans*, it acts in a hexa­dentate fashion. In this case, the formation of a dinuclear complex with a *μ*-*N*,*N* bridge is generally observed, for example in a dinuclear copper(II) complex (Dragancea *et al.*, 2014 ▸). The *S-cis–enol* configuration leads to the formation of square-grid complexes by directed self-assembly (Bikas *et al.*, 2015 ▸; Sow *et al.*, 2013 ▸; Li *et al.*, 2014 ▸). In these complexes, μ-*O* and μ-*N*,*N* atoms bridge the metal ions, which display N_4_O_2_ or N_5_O octa­hedral environments (Shuvaev *et al.*, 2010 ▸).
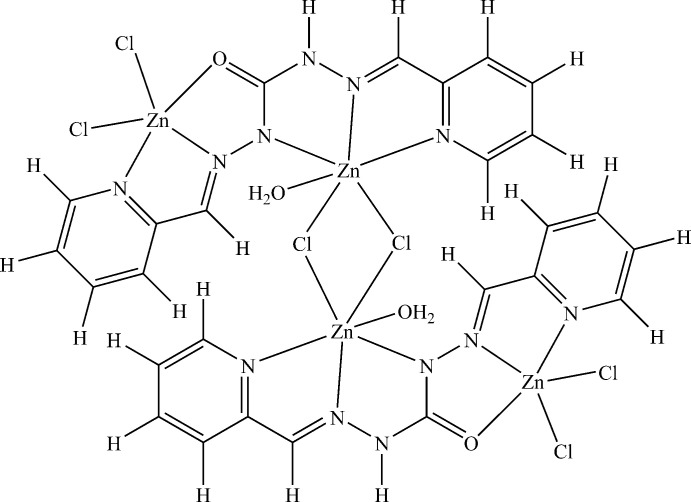


The behavior of these mol­ecules has attracted the inter­est of chemists working in coordination chemistry. The free dicarbonohydrazide exhibits biological activities (Bacchi *et al.*, 1999 ▸; Kothari & Sharma, 2010 ▸), which are increased upon complexation with certain transition metals (Wu *et al.*, 2009 ▸; Bikas *et al.*, 2015 ▸). The synthesis of high nuclearity complexes of transition metals derived from these types of ligands are highly targeted because of their magnetic (Sow *et al.*, 2013 ▸; Zhang *et al.*, 2014*a* ▸; Dragancea *et al.*, 2014 ▸), catalytic (Bikas *et al.*, 2015 ▸), biological (Zhang *et al.*, 2014 ▸) and optical (Easwaran potti *et al.*, 2007 ▸) properties. Recently, our research group synthesized a new tetra­nuclear grid complex [Zn_4_(H*L* ^1^)_4_](NO_3_)_4_·2H_2_O where H_2_ *L* ^1^ is 1,5-bis­[1-(pyridin-2-yl)ethyl­idene)carbonohydrazide]. The study of the fluorescence properties of the ligand H_2_ *L* ^1^ and its complex revealed that complexation increased the fluorescent properties of the ligand (Seck *et al.*, 2018 ▸). In a continuation of our work on symmetrical dicarbonohydrazide ligands, we have synthesized and characterized a new tetra­nuclear zinc(II) complex formulated as {[Zn_2_(HL)(H_2_O)(Cl_2_)](μCl)_2_[Zn_2_(HL)(H_2_O)(Cl)]}_2_ where H_2_ *L* is 1,5-bis­(pyridin-2-yl­methyl­ene)carbono­hydrazide.

## Structural commentary   

**Figure 1:**
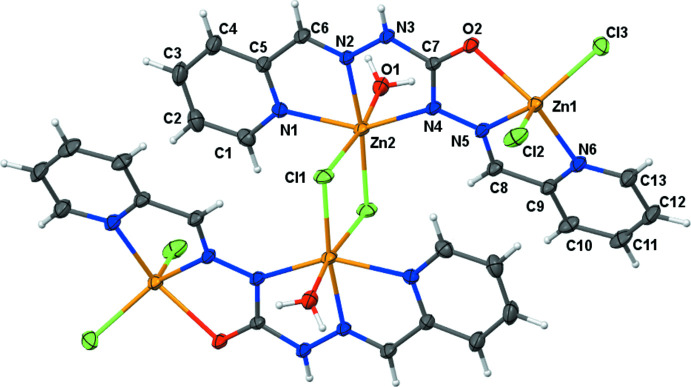
A view of the title compound, showing the atom-numbering scheme. Displacement ellipsoids are plotted at the 30% probability level. Unlabelled atoms are generated by the symmetry operation 1 − *x*, 1 − *y*, −*z*.

The title compound is a centrosymmetric tetra­nuclear Zn^II^ complex composed by two dinuclear entities. Each dinuclear entity contains one ligand mol­ecule acting in monodeprotonated form, three bonded chloride anions, one bonded water mol­ecule, and two Zn^II^ cations. The two units are linked by two choride anions acting as bridges (Fig. 1 ▸). Each monodeprotonated organic mol­ecule acts through two azomethine nitro­gen atoms, two pyridine nitro­gen atoms, one hydrazinyl nitro­gen atom and one carbonyl oxygen atom, resulting in a hexa­dentate ligand. The Zn1 and Zn2 cations are situated, respectively in N_2_OCl_2_ and N_3_OCl_2_ coordination sites (Fig. 1 ▸). In the structure of the complex, the two ligand mol­ecules are arranged in the *Z–E* form.

The Zn1 atom is penta­coordinated by one pyridine nitro­gen atom, one azomethine nitro­gen atom, one oxygen atom, and two terminal chloride anions. According to the Addison (1984 ▸) index, the coordination geometry around a penta­coordinated metal center can be discussed in terms of the τ parameter [defined as τ = (*β* - *α*)/60 where *β* and *α* are the largest values of the bond angles around the central atom]; *τ* = 0 for a perfect square pyramidal geometry while *τ* = 1 for a perfect trigonal–bipyramidal geometry. In the case of the title complex, the *τ* value of 0.1085 is indicative of a distorted square-pyramidal geometry around the Zn1 center. The equatorial plane is occupied by atoms N5, N6, Cl3, O2 while the apical position is occupied by Cl2. The angles N5—Zn1—O2 [72.76 (9)°], O2—Zn1—Cl3 [96.00 (6)°], Cl3—Zn1—N6 [97.10 (8)°] and N6—Zn1—N5 [75.82 (10)°] deviate from those for a regular square pyramid. The *transoid* angles in the basal plane O2—Zn1—N6 and N5—Zn1—Cl3 deviate severely from linearity with values of 144.87 (10) and 138.36 (8)°, respectively (Table 1 ▸). The angles involving the atoms in the axial position deviate severely from the ideal value of 90°, being in the range 97.45 (7)–110.85 (8)°.

The geometry around the hexa­coordinated Zn2 atom is best described as distorted octahedral. The basal plane is occupied by atoms N2, N4, N1 and Cl1 with *cissoid* bond angles in the range 73.95 (9)–111.20 (7)° and *transoid* angles of 171.67 (8)° [N2—Zn2—Cl1] and 148.24 (10)° [N4—Zn2—N1]. The sum of the angles subtended by the atoms in the plane is 359.77°. The apical positions are occupied by O1 and Cl1^i^ with O 1—Zn2—Cl1^i^ = 172.20 (7)° (Table 1 ▸). The deviation of the angles around the Zn2 cation with respect to the valence angles for a regular octa­hedron (180 and 90°) indicates that the geometry around the Zn2 ion is a distorted octa­hedron (Fig. 1 ▸). The five-membered rings (NCNNZn and NCCNZn) formed by the ligand with Zn2 impose large distortions on the ideal angles of a regular octa­hedron with bite angles in the range 73.95 (9)–74.29 (9)°.

The Zn2—Cl1—Zn2^i^ angle of 92.37 (3)° is in accordance with the value reported for the complex di-μ-chlorido-bis­{[2-({[2-(2-pyrid­yl)eth­yl](2-pyridyl­meth­yl)amino}­meth­yl)-phen­ol]zinc(II)} bis­(perchlorate) dihydrate (Coelho *et al.*, 2010 ▸). The zinc–halogen distances Zn2—Cl1 and Zn2^i^—Cl1 of 2.2873 (8) and 2.7489 (10) Å, respectively, agree with those for a chloride anion in a bridging position (Coelho *et al.*, 2010 ▸; Yu *et al.*, 2009 ▸). The distances Zn1—Cl2 and Zn1—Cl3 of 2.2573 (10) and 2.2477 (9) Å, respectively, are indicative of a unidentate terminal chloride anion (Sanyal *et al.*, 2014 ▸).

Only one weak intra­molecular C—H⋯O hydrogen bond (Table 2 ▸) occurs.

## Supra­molecular features   

**Figure 2:**
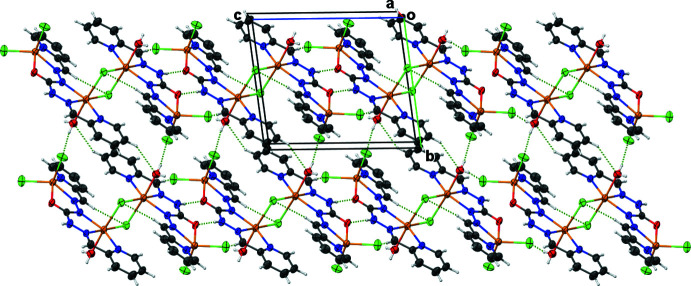
View of the chains formed by hydrogen bonds in the *ac* plane.

In the crystal, numerous inter­molecular O—H⋯Cl, C—H⋯O, C—H⋯Cl and N—H⋯O hydrogen bonds are observed (Fig. 2 ▸, Table 2 ▸). An N—H⋯O type occurs between the oxygen atom O2 of the ligand, which acts as a proton acceptor, and the nitro­gen atom of the hydrazinyl group, which acts as the proton donor. An O—H⋯Cl link is established between a water mol­ecule in the apical position of the Zn2 ion, acting as proton donor, and a terminal chloride ions linked to Zn1 as proton acceptor. These inter­molecular hydrogen bonds ensure the cohesion of the crystal, developing a planar two-dimensional structure in the *ac* plane.

## Database survey   

A survey of the Cambridge Structural Database (CSD, Version 5.40, October 2019; Groom *et al.*, 2016 ▸) reveals five examples of crystal structures containing H_2_ *L* derivatives where the mol­ecule is monoprotonated (H_3_ *L* ^+^) or diprotonated (H_4_ *L* ^2+^) and additionally one Dy complex mol­ecule in which H*L* ^−^ and *L* ^2−^ are present as ligands. Among the diprotonated mol­ecules, three different counter-ions are present: I^−^ in AVOSOV (Hoque *et al.*, 2016 ▸), ClO_4_^−^ in LOFDUH (Hoque *et al.*, 2014 ▸), and SO_4_^2−^ in LOFFAP (Hoque *et al.*, 2014 ▸) and LOFFAP01 (Hoque *et al.*, 2016 ▸). In the structure incorporating monoprotonated H_3_ *L* ^+^, H_2_PO_4_^−^ is the counter-ion (LOFFIX; Hoque *et al.*, 2014 ▸). The tetra­nuclear Dy^3+^ complex has a [2 x 2] grid structure (DIGQER; Randell *et al.*, 2013 ▸).

## Synthesis and crystallization   

**Synthesis of the H_2_** ***L*** **ligand**

Carbonohydrazide (2 g, 22.2 mmol) was introduced into a 100 mL flask containing 20 mL of methanol. To the resulting suspension was added a methano­lic solution containing 2-pyridine­carbaldehyde (4.757 g 44.4 mmol) and two drops of glacial acetic acid. The mixture was stirred under reflux for 2 h. After being kept for two days at 277 K, the resulting orange solution yielded a precipitate, which was recovered by filtration. The solid was washed successively with cold methanol (2 × 10 mL) and diethyl ether (2 × 10 mL) before being dried under P_2_O_5_; m.p. 489 K, yield 82%. Analysis calculated for [C_13_H_12_N_6_O] C, 58.20; H, 4.51; N, 31.33. Found: C, 58.17; H, 4.49; N, 31.30. IR (cm^−1^): 3439, 3204, 3198, 3055, 2936, 1684, 1582, 1610, 1582, 1532, 1467, 1360, 1274, 1131. ^1^H NMR (DMSO-*d*_6_, δ in ppm): 7.6–8.72 (*m*, 8H, H_Py_); 10.82 (*s*, 2H, H—N); 8.03 (*s*, 2H, H—C=N). ^13^C NMR (DMSO-*d*_6_, δ in ppm): 157.9 (C=O); 154.70 (C_Py_); 148.07 (C_Py_); 146.67 (C=N) imine; 137.60 (CPy); 123.00 (C_Py_); 119.09 (C_Py_).

**Synthesis of the title complex**

The title complex was prepared by mixing a solution of H_2_ *L* (134.15 mg, 0.5 mmol) in 10 mL of methanol and a methano­lic solution of ZnCl_2_ (68.15 mg, 0.5 mmol). A yellow solution was obtained after stirring for 1 h at room temperature. The solution was filtered, and the filtrate left for slow evaporation. After two weeks, yellow crystals suitable for X-ray diffraction were collected, yield 87.9%. Analysis calculated for [C_26_H_26_Cl_6_Zn_4_N_12_O_4_] C, 29.89; H, 2.51; N, 16.09. Found: C, 29.88; H, 2.49; N, 16.05. Λ_M_ (S cm^2^ mol^−^): 11. IR (cm^−1^): 3428, 3116, 3043, 1585, 1553, 1497, 1461, 1377, 1313, 1226, 1143, 820.

## Refinement   

Crystal data, data collection and structure refinement details are summarized in Table 3 ▸. N- and C-bound H atoms were refined with *U*_iso_(H) = 1.2*U*_eq_(N) or 1.5*U*_eq_(O). C atoms were placed in calculated positions and refined as riding with C—H = 0.93 Å and *U*_iso_(H) = 1.2*U*_eq_(C).

## Supplementary Material

Crystal structure: contains datablock(s) I. DOI: 10.1107/S2056989020009834/ex2035sup1.cif

Structure factors: contains datablock(s) I. DOI: 10.1107/S2056989020009834/ex2035Isup2.hkl

CCDC reference: 2011426

Additional supporting information:  crystallographic information; 3D view; checkCIF report

## Figures and Tables

**Table 1:** Selected geometric parameters (Å, °)

Zn1—N5	2.069 (2)	Zn2—O1	2.132 (3)
Zn1—N6	2.191 (3)	Zn2—N4	2.139 (3)
Zn1—O2	2.237 (2)	Zn2—N1	2.184 (3)
Zn1—Cl3	2.2477 (9)	Zn2—Cl1	2.2873 (8)
Zn1—Cl2	2.2573 (10)	Zn2—Cl1^i^	2.7489 (10)
Zn2—N2	2.117 (2)		
			
N5—Zn1—N6	75.82 (10)	N2—Zn2—N1	74.29 (9)
N5—Zn1—O2	72.76 (9)	O1—Zn2—N1	87.40 (10)
N6—Zn1—O2	144.87 (10)	N4—Zn2—N1	148.24 (10)
N5—Zn1—Cl3	138.36 (8)	N2—Zn2—Cl1	171.67 (8)
N6—Zn1—Cl3	97.10 (8)	O1—Zn2—Cl1	95.98 (8)
O2—Zn1—Cl3	96.00 (6)	N4—Zn2—Cl1	111.20 (7)
N5—Zn1—Cl2	110.85 (8)	N1—Zn2—Cl1	100.33 (7)
N6—Zn1—Cl2	108.19 (9)	N2—Zn2—Cl1^i^	85.59 (8)
O2—Zn1—Cl2	97.45 (7)	O1—Zn2—Cl1^i^	172.20 (7)
Cl3—Zn1—Cl2	110.31 (4)	N4—Zn2—Cl1^i^	92.27 (8)
N2—Zn2—O1	90.19 (11)	N1—Zn2—Cl1^i^	85.15 (8)
N2—Zn2—N4	73.95 (9)	Cl1—Zn2—Cl1^i^	87.63 (3)
O1—Zn2—N4	92.86 (10)	Zn2—Cl1—Zn2^i^	92.37 (3)

Symmetry code: (i) 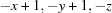.

**Table 2:** Hydrogen-bond geometry (Å, °)

*D*—H⋯*A*	*D*—H	H⋯*A*	*D*⋯*A*	*D*—H⋯*A*
O1—H1*A*⋯Cl3^ii^	0.82 (2)	2.27 (2)	3.052 (3)	161 (5)
O1—H1*B*⋯Cl2^iii^	0.82 (2)	2.32 (2)	3.129 (3)	169 (5)
C8—H8⋯Cl1	0.93	2.82	3.649 (3)	149
C2—H2⋯O1^iv^	0.93	2.50	3.342 (4)	151
C6—H6⋯Cl3^v^	0.93	2.55	3.425 (3)	158
N3—H3*N*⋯O2^v^	0.85 (4)	2.00 (4)	2.837 (3)	170 (4)

Symmetry codes: (ii) 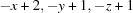; (iii) 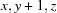; (iv) 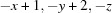; (v) 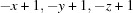.

**Table 3:** Experimental details

Crystal data	
Chemical formula	[Zn_4_(C_13_H_11_N_6_O)_2_Cl_6_(H_2_O)_2_]
*M*_r_	1044.77
Crystal system, space group	Triclinic, *P* 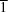
Temperature (K)	293
*a*, *b*, *c* (Å)	9.2002 (4), 9.4306 (4), 11.7651 (4)
α, β, γ (°)	94.639 (3), 110.091 (4), 97.599 (3)
*V* (Å^3^)	941.47 (7)
*Z*	1
Radiation type	Mo *K*α
μ (mm^−1^)	2.99
Crystal size (mm)	0.21 × 0.10 × 0.05

Data collection	
Diffractometer	XtaLAB AFC12 (RINC): Kappa single
Absorption correction	Multi-scan (*CrysAlis PRO*; Rigaku OD, 2018 ▸)
*T*_min_, *T*_max_	0.375, 1.000
No. of measured, independent and observed [*I* > 2σ(*I*)] reflections	15934, 4132, 3343
*R*_int_	0.049
(sin θ/λ)_max_ (Å^−1^)	0.641

Refinement	
*R*[*F* ^2^ > 2σ(*F* ^2^)], *wR*(*F* ^2^), *S*	0.039, 0.103, 1.05
No. of reflections	4127
No. of parameters	244
No. of restraints	2
H-atom treatment	H atoms treated by a mixture of independent and constrained refinement
Δρ_max_, Δρ_min_ (e Å^−3^)	0.80, −0.94

Computer programs: *CrysAlis PRO* (Rigaku OD, 2018 ▸), *SHELXT* (Sheldrick, 2015*a* ▸), *SHELXL2018/3* (Sheldrick, 2015*b* ▸) and *ORTEP-3 for Windows* (Farrugia, 2012 ▸).

## supplementary crystallographic information

### Crystal data

**Table:**

[Zn_4_(C_13_H_11_N_6_O)_2_Cl_6_(H_2_O)_2_]	*Z* = 1
*M**_r_* = 1044.77	*F*(000) = 520
Triclinic, *P*1	*D*_x_ = 1.843 Mg m^−^^3^
*a* = 9.2002 (4) Å	Mo *K*α radiation, λ = 0.71073 Å
*b* = 9.4306 (4) Å	Cell parameters from 6440 reflections
*c* = 11.7651 (4) Å	θ = 4.3–30.7°
α = 94.639 (3)°	µ = 2.99 mm^−^^1^
β = 110.091 (4)°	*T* = 293 K
γ = 97.599 (3)°	Tab, pale yellow
*V* = 941.47 (7) Å^3^	0.21 × 0.10 × 0.05 mm

### Data collection

**Table:**

XtaLAB AFC12 (RINC): Kappa single diffractometer	4132 independent reflections
Radiation source: micro-focus sealed X-ray tube, Rigaku (Mo) X-ray Source	3343 reflections with *I* > 2σ(*I*)
Mirror monochromator	*R*_int_ = 0.049
ω scans	θ_max_ = 27.1°, θ_min_ = 4.4°
Absorption correction: multi-scan (CrysAlisPro; Rigaku OD, 2018)	*h* = −11→11
*T*_min_ = 0.375, *T*_max_ = 1.000	*k* = −12→12
15934 measured reflections	*l* = −15→15

### Refinement

**Table:**

Refinement on *F*^2^	Primary atom site location: dual
Least-squares matrix: full	Secondary atom site location: difference Fourier map
*R*[*F*^2^ > 2σ(*F*^2^)] = 0.039	Hydrogen site location: mixed
*wR*(*F*^2^) = 0.103	H atoms treated by a mixture of independent and constrained refinement
*S* = 1.05	*w* = 1/[σ^2^(*F*_o_^2^) + (0.0583*P*)^2^ + 0.5038*P*] where *P* = (*F*_o_^2^ + 2*F*_c_^2^)/3
4127 reflections	(Δ/σ)_max_ < 0.001
244 parameters	Δρ_max_ = 0.80 e Å^−^^3^
2 restraints	Δρ_min_ = −0.94 e Å^−^^3^

### Special details

**Table:**

Geometry. All esds (except the esd in the dihedral angle between two l.s. planes) are estimated using the full covariance matrix. The cell esds are taken into account individually in the estimation of esds in distances, angles and torsion angles; correlations between esds in cell parameters are only used when they are defined by crystal symmetry. An approximate (isotropic) treatment of cell esds is used for estimating esds involving l.s. planes.

### Fractional atomic coordinates and isotropic or equivalent isotropic displacement parameters (Å^2^)

**Table:**

	*x*	*y*	*z*	*U*_iso_*/*U*_eq_	
Zn1	0.80255 (4)	0.26414 (4)	0.42860 (3)	0.03096 (12)	
Zn2	0.57362 (4)	0.62337 (4)	0.14274 (3)	0.03302 (12)	
Cl1	0.66995 (10)	0.59565 (10)	−0.01140 (7)	0.0390 (2)	
Cl2	0.61634 (11)	0.06523 (11)	0.34885 (11)	0.0537 (3)	
Cl3	0.93484 (10)	0.25785 (12)	0.62765 (8)	0.0500 (3)	
O1	0.7387 (3)	0.8113 (3)	0.2417 (2)	0.0427 (6)	
H1A	0.823 (3)	0.802 (5)	0.291 (4)	0.064*	
H1B	0.718 (6)	0.878 (4)	0.278 (4)	0.064*	
N1	0.4062 (3)	0.7669 (3)	0.0700 (2)	0.0312 (6)	
N4	0.6695 (3)	0.4927 (3)	0.2813 (2)	0.0316 (6)	
N5	0.7847 (3)	0.4085 (3)	0.3030 (2)	0.0290 (5)	
O2	0.6460 (3)	0.4146 (3)	0.4577 (2)	0.0366 (5)	
N2	0.4518 (3)	0.6419 (3)	0.2652 (2)	0.0297 (5)	
C5	0.3207 (4)	0.7960 (4)	0.1389 (3)	0.0345 (7)	
C7	0.6080 (4)	0.4865 (3)	0.3697 (3)	0.0291 (6)	
N3	0.4932 (3)	0.5689 (3)	0.3617 (3)	0.0362 (7)	
H3N	0.442 (5)	0.567 (4)	0.409 (4)	0.043*	
C1	0.3864 (4)	0.8307 (4)	−0.0303 (3)	0.0397 (8)	
H1	0.444294	0.809996	−0.078687	0.048*	
C10	1.1160 (4)	0.3293 (4)	0.2278 (4)	0.0423 (8)	
H10	1.115666	0.386847	0.167216	0.051*	
N6	0.9966 (3)	0.2407 (3)	0.3647 (3)	0.0359 (6)	
C9	1.0000 (4)	0.3243 (4)	0.2777 (3)	0.0325 (7)	
C4	0.2147 (5)	0.8905 (4)	0.1096 (4)	0.0481 (9)	
H4	0.156102	0.908579	0.158053	0.058*	
C8	0.8767 (4)	0.4134 (4)	0.2430 (3)	0.0327 (7)	
H8	0.866238	0.470241	0.180546	0.039*	
C2	0.2826 (5)	0.9266 (4)	−0.0646 (3)	0.0480 (9)	
H2	0.270608	0.969450	−0.135049	0.058*	
C11	1.2334 (5)	0.2466 (5)	0.2698 (4)	0.0510 (10)	
H11	1.312414	0.247754	0.237298	0.061*	
C6	0.3499 (4)	0.7248 (4)	0.2481 (3)	0.0385 (8)	
H6	0.297366	0.738940	0.301839	0.046*	
C3	0.1979 (5)	0.9572 (5)	0.0069 (4)	0.0544 (10)	
H3	0.129191	1.022847	−0.013639	0.065*	
C13	1.1100 (4)	0.1625 (4)	0.4044 (4)	0.0459 (9)	
H13	1.107789	0.104825	0.464524	0.055*	
C12	1.2309 (5)	0.1637 (5)	0.3596 (4)	0.0522 (10)	
H12	1.309318	0.108995	0.390059	0.063*	

### Atomic displacement parameters (Å^2^)

**Table:**

	*U*^11^	*U*^22^	*U*^33^	*U*^12^	*U*^13^	*U*^23^
Zn1	0.0313 (2)	0.0364 (2)	0.0331 (2)	0.01189 (15)	0.01808 (15)	0.00942 (15)
Zn2	0.0394 (2)	0.0418 (2)	0.0306 (2)	0.01887 (17)	0.02281 (16)	0.01138 (16)
Cl1	0.0456 (5)	0.0475 (5)	0.0364 (4)	0.0111 (4)	0.0291 (4)	0.0070 (3)
Cl2	0.0430 (5)	0.0450 (5)	0.0757 (7)	0.0007 (4)	0.0311 (5)	−0.0103 (5)
Cl3	0.0343 (4)	0.0852 (7)	0.0373 (4)	0.0232 (5)	0.0152 (4)	0.0142 (4)
O1	0.0397 (14)	0.0429 (15)	0.0450 (15)	0.0176 (12)	0.0110 (11)	0.0047 (11)
N1	0.0327 (13)	0.0373 (15)	0.0285 (13)	0.0097 (11)	0.0152 (11)	0.0078 (11)
N4	0.0349 (14)	0.0408 (15)	0.0309 (13)	0.0187 (12)	0.0209 (11)	0.0106 (11)
N5	0.0287 (13)	0.0330 (14)	0.0329 (13)	0.0123 (11)	0.0173 (11)	0.0077 (11)
O2	0.0470 (13)	0.0480 (14)	0.0323 (12)	0.0270 (11)	0.0263 (10)	0.0183 (10)
N2	0.0337 (13)	0.0371 (14)	0.0275 (12)	0.0163 (11)	0.0175 (11)	0.0101 (11)
C5	0.0331 (16)	0.0419 (18)	0.0329 (16)	0.0152 (14)	0.0136 (13)	0.0077 (14)
C7	0.0296 (15)	0.0359 (17)	0.0279 (15)	0.0123 (13)	0.0155 (12)	0.0047 (12)
N3	0.0420 (16)	0.0511 (18)	0.0326 (14)	0.0273 (14)	0.0251 (12)	0.0166 (13)
C1	0.0441 (19)	0.043 (2)	0.0340 (17)	0.0059 (16)	0.0165 (15)	0.0098 (15)
C10	0.0406 (19)	0.047 (2)	0.052 (2)	0.0100 (16)	0.0311 (17)	0.0059 (16)
N6	0.0330 (14)	0.0446 (16)	0.0402 (15)	0.0168 (12)	0.0212 (12)	0.0092 (12)
C9	0.0285 (15)	0.0394 (17)	0.0359 (16)	0.0083 (13)	0.0188 (13)	0.0046 (13)
C4	0.046 (2)	0.056 (2)	0.052 (2)	0.0282 (19)	0.0211 (18)	0.0166 (18)
C8	0.0348 (16)	0.0367 (17)	0.0377 (17)	0.0136 (14)	0.0230 (14)	0.0102 (13)
C2	0.049 (2)	0.052 (2)	0.043 (2)	0.0120 (18)	0.0111 (17)	0.0210 (17)
C11	0.0386 (19)	0.057 (2)	0.070 (3)	0.0108 (18)	0.0369 (19)	0.001 (2)
C6	0.0401 (18)	0.053 (2)	0.0368 (17)	0.0227 (16)	0.0247 (15)	0.0124 (15)
C3	0.049 (2)	0.057 (3)	0.062 (3)	0.029 (2)	0.0153 (19)	0.023 (2)
C13	0.046 (2)	0.054 (2)	0.051 (2)	0.0236 (18)	0.0266 (17)	0.0155 (18)
C12	0.040 (2)	0.065 (3)	0.065 (3)	0.0294 (19)	0.0281 (19)	0.011 (2)

### Geometric parameters (Å, º)

**Table:**

Zn1—N5	2.069 (2)	C7—N3	1.374 (4)
Zn1—N6	2.191 (3)	N3—H3N	0.85 (4)
Zn1—O2	2.237 (2)	C1—C2	1.384 (5)
Zn1—Cl3	2.2477 (9)	C1—H1	0.9300
Zn1—Cl2	2.2573 (10)	C10—C9	1.381 (4)
Zn2—N2	2.117 (2)	C10—C11	1.391 (5)
Zn2—O1	2.132 (3)	C10—H10	0.9300
Zn2—N4	2.139 (3)	N6—C13	1.333 (4)
Zn2—N1	2.184 (3)	N6—C9	1.348 (4)
Zn2—Cl1	2.2873 (8)	C9—C8	1.465 (4)
Zn2—Cl1^i^	2.7489 (10)	C4—C3	1.378 (5)
O1—H1A	0.816 (19)	C4—H4	0.9300
O1—H1B	0.819 (19)	C8—H8	0.9300
N1—C1	1.335 (4)	C2—C3	1.367 (6)
N1—C5	1.346 (4)	C2—H2	0.9300
N4—C7	1.345 (4)	C11—C12	1.369 (6)
N4—N5	1.374 (3)	C11—H11	0.9300
N5—C8	1.274 (4)	C6—H6	0.9300
O2—C7	1.258 (4)	C3—H3	0.9300
N2—C6	1.273 (4)	C13—C12	1.385 (5)
N2—N3	1.344 (3)	C13—H13	0.9300
C5—C4	1.382 (5)	C12—H12	0.9300
C5—C6	1.457 (4)		
			
N5—Zn1—N6	75.82 (10)	C4—C5—C6	122.1 (3)
N5—Zn1—O2	72.76 (9)	O2—C7—N4	126.6 (3)
N6—Zn1—O2	144.87 (10)	O2—C7—N3	117.8 (3)
N5—Zn1—Cl3	138.36 (8)	N4—C7—N3	115.6 (3)
N6—Zn1—Cl3	97.10 (8)	N2—N3—C7	116.7 (3)
O2—Zn1—Cl3	96.00 (6)	N2—N3—H3N	120 (3)
N5—Zn1—Cl2	110.85 (8)	C7—N3—H3N	123 (3)
N6—Zn1—Cl2	108.19 (9)	N1—C1—C2	122.3 (3)
O2—Zn1—Cl2	97.45 (7)	N1—C1—H1	118.9
Cl3—Zn1—Cl2	110.31 (4)	C2—C1—H1	118.9
N2—Zn2—O1	90.19 (11)	C9—C10—C11	118.9 (3)
N2—Zn2—N4	73.95 (9)	C9—C10—H10	120.6
O1—Zn2—N4	92.86 (10)	C11—C10—H10	120.6
N2—Zn2—N1	74.29 (9)	C13—N6—C9	118.7 (3)
O1—Zn2—N1	87.40 (10)	C13—N6—Zn1	128.7 (2)
N4—Zn2—N1	148.24 (10)	C9—N6—Zn1	112.5 (2)
N2—Zn2—Cl1	171.67 (8)	N6—C9—C10	121.8 (3)
O1—Zn2—Cl1	95.98 (8)	N6—C9—C8	115.8 (3)
N4—Zn2—Cl1	111.20 (7)	C10—C9—C8	122.3 (3)
N1—Zn2—Cl1	100.33 (7)	C3—C4—C5	118.5 (4)
N2—Zn2—Cl1^i^	85.59 (8)	C3—C4—H4	120.8
O1—Zn2—Cl1^i^	172.20 (7)	C5—C4—H4	120.8
N4—Zn2—Cl1^i^	92.27 (8)	N5—C8—C9	116.3 (3)
N1—Zn2—Cl1^i^	85.15 (8)	N5—C8—H8	121.9
Cl1—Zn2—Cl1^i^	87.63 (3)	C9—C8—H8	121.9
Zn2—Cl1—Zn2^i^	92.37 (3)	C3—C2—C1	118.7 (3)
Zn2—O1—H1A	119 (3)	C3—C2—H2	120.6
Zn2—O1—H1B	124 (4)	C1—C2—H2	120.6
H1A—O1—H1B	101 (5)	C12—C11—C10	119.1 (3)
C1—N1—C5	118.6 (3)	C12—C11—H11	120.4
C1—N1—Zn2	127.0 (2)	C10—C11—H11	120.4
C5—N1—Zn2	114.4 (2)	N2—C6—C5	115.8 (3)
C7—N4—N5	108.9 (2)	N2—C6—H6	122.1
C7—N4—Zn2	116.75 (19)	C5—C6—H6	122.1
N5—N4—Zn2	134.33 (19)	C2—C3—C4	119.9 (3)
C8—N5—N4	120.9 (3)	C2—C3—H3	120.1
C8—N5—Zn1	119.3 (2)	C4—C3—H3	120.1
N4—N5—Zn1	119.75 (19)	N6—C13—C12	122.5 (4)
C7—O2—Zn1	109.38 (18)	N6—C13—H13	118.7
C6—N2—N3	123.3 (3)	C12—C13—H13	118.7
C6—N2—Zn2	119.7 (2)	C11—C12—C13	119.0 (3)
N3—N2—Zn2	116.96 (18)	C11—C12—H12	120.5
N1—C5—C4	122.1 (3)	C13—C12—H12	120.5
N1—C5—C6	115.8 (3)		
			
C7—N4—N5—C8	−169.4 (3)	C13—N6—C9—C8	−176.8 (3)
Zn2—N4—N5—C8	10.8 (5)	Zn1—N6—C9—C8	0.1 (4)
C7—N4—N5—Zn1	13.8 (3)	C11—C10—C9—N6	−0.7 (6)
Zn2—N4—N5—Zn1	−166.03 (17)	C11—C10—C9—C8	176.9 (4)
C1—N1—C5—C4	−0.6 (5)	N1—C5—C4—C3	−0.5 (6)
Zn2—N1—C5—C4	176.8 (3)	C6—C5—C4—C3	177.8 (4)
C1—N1—C5—C6	−179.0 (3)	N4—N5—C8—C9	177.2 (3)
Zn2—N1—C5—C6	−1.6 (4)	Zn1—N5—C8—C9	−6.0 (4)
Zn1—O2—C7—N4	−10.3 (4)	N6—C9—C8—N5	3.7 (5)
Zn1—O2—C7—N3	170.3 (2)	C10—C9—C8—N5	−174.0 (3)
N5—N4—C7—O2	−1.0 (5)	N1—C1—C2—C3	0.2 (6)
Zn2—N4—C7—O2	178.8 (3)	C9—C10—C11—C12	−0.4 (6)
N5—N4—C7—N3	178.4 (3)	N3—N2—C6—C5	179.5 (3)
Zn2—N4—C7—N3	−1.7 (4)	Zn2—N2—C6—C5	2.9 (4)
C6—N2—N3—C7	−178.3 (3)	N1—C5—C6—N2	−0.7 (5)
Zn2—N2—N3—C7	−1.6 (4)	C4—C5—C6—N2	−179.1 (4)
O2—C7—N3—N2	−178.4 (3)	C1—C2—C3—C4	−1.3 (7)
N4—C7—N3—N2	2.2 (5)	C5—C4—C3—C2	1.5 (7)
C5—N1—C1—C2	0.8 (5)	C9—N6—C13—C12	−0.1 (6)
Zn2—N1—C1—C2	−176.2 (3)	Zn1—N6—C13—C12	−176.5 (3)
C13—N6—C9—C10	0.9 (5)	C10—C11—C12—C13	1.2 (7)
Zn1—N6—C9—C10	177.9 (3)	N6—C13—C12—C11	−1.0 (7)

Symmetry code: (i) −*x*+1, −*y*+1, −*z*.

### Hydrogen-bond geometry (Å, º)

**Table:**

*D*—H···*A*	*D*—H	H···*A*	*D*···*A*	*D*—H···*A*
O1—H1*A*···Cl3^ii^	0.82 (2)	2.27 (2)	3.052 (3)	161 (5)
O1—H1*B*···Cl2^iii^	0.82 (2)	2.32 (2)	3.129 (3)	169 (5)
C8—H8···Cl1	0.93	2.82	3.649 (3)	149
C2—H2···O1^iv^	0.93	2.50	3.342 (4)	151
C6—H6···Cl3^v^	0.93	2.55	3.425 (3)	158
N3—H3*N*···O2^v^	0.85 (4)	2.00 (4)	2.837 (3)	170 (4)

Symmetry codes: (ii) −*x*+2, −*y*+1, −*z*+1; (iii) *x*, *y*+1, *z*; (iv) −*x*+1, −*y*+2, −*z*; (v) −*x*+1, −*y*+1, −*z*+1.
